# SWOT Analysis of Total Sanitation Campaign in Yavatmal District of Maharashtra

**DOI:** 10.4103/0970-0218.43233

**Published:** 2008-10

**Authors:** Geeta Pardeshi, Avinash Shirke, Minal Jagtap

**Affiliations:** Department of Preventive and Social Medicine, Government Medical College, Akola, India

**Keywords:** Total Sanitation Campaign, SWOT analysis

## Abstract

**Aims:**

To study the strengths, weaknesses, opportunities, and threats of the Total Sanitation Campaign (TSC) in the Yavatmal district of Maharashtra.

**Methodology:**

Data was collected in December 2006 through interviews with stakeholders, house-to-house surveys, focus group discussions, and transect walks. Information in each category was finalized in a meeting after brainstorming and discussion with the TSC cell members.

**Results:**

The strengths of the campaign were innovations in Information Education and Communication, motivation through incentives, competitive spirit, active participation and partnerships, involvement of women, and universal coverage. The main weaknesses of the program were the absence of Rural Sanitary Marts/Production Centers, poor maintenance of Women Sanitary Complexes, lack of facilities for monitoring/ follow-up and a temporary focus of the campaign approach. There is an opportunity to tap additional resources, learn from other experiences, and institute back-up agencies to support and guide the community in the post-TSC phase. A change in administration and local leadership and loss of priority and interest needed to sustain the momentum while scaling up the interventions are possible threats for the program.

## Introduction

Sanitation is a basic necessity that affects everyone's life. Proper disposal of household waste is important to prevent feco-oral and vector borne diseases.(1,2) As per the 2001 census, only 22% of households in rural India have access to household latrines.(3)

The Central Rural Sanitation Program (CRSP) launched in 1986 and revised in 1992 was a traditional, supply-driven subsidy-oriented program. In April 1999, CRSP was restructured and launched as the Total Sanitation Campaign (TSC) making it ‘people oriented’ and ‘demand driven’. The program is implemented in a campaign mode with the district as a unit.

The TSC in Yavatmal was approved in March 2001. The startup activities included a baseline survey, preparation of plans, capacity building, and human resource development. The key intervention areas were the construction of individual household latrines, school sanitation, and anganwadi toilets. The Nirmal Gram Puraskar Yojana initiated by the Central Government was implemented in the district. The criteria for Nirmal Gram awards included (a) 100% sanitation coverage with individual/community latrines, (b) 100% school and anganwadi sanitation coverage, (c) a living environment free from open defecation, and (d) clean environment maintenance. The villages were assessed by an independent agency after which they were declared eligible for the awards. A total of nine Grampanchayats achieved the Nirmal Gram status in March 2006 and in October 2006 another 110 Grampanchayats submitted their proposals for the award.

## Methodology

Quantitative and rapid appraisal methods were used to collect data. The field work for the study was done in December 2006 in 4 phases.

**Interview of key informants:** Data was collected through review of records and interviews of stakeholders viz. members of the Total Sanitation Cell and key village informants.

**House-to-house surveys:** House-to-house surveys were conducted in 4 Nirmal Gram villages and 4 control villages to assess the field situation. These villages were selected by simple random sampling using the lottery method from the list of nine Nirmal Gram villages. The control villages were selected from the respective talukas with approximately similar populations. A pre-tested semi open-ended questionnaire was used to collect data regarding different methods of disposal of household waste. In Nirmal Gram villages, 416/490 households (85%) and in the control villages 535/620 (86%) households were covered during the survey. Analysis was done by calculating proportions.

**Transect walks:** Transect walks were conducted for on-field observations and discussions with the villagers at selected transect points. A community resource map was drawn and a route was selected to cover important transect points. The transect points selected were individual household latrines, community latrines, school latrines, anganwadi latrines, sullage and solid waste disposal facilities, animal waste disposal, etc. All the information was analyzed and recorded in a tabular format.

**Triangulation and discussions:** All data collected in the three phases were triangulated, analyzed and presented in a meeting of the investigators with the TSC cell members. After discussions, the points in each category of the SWOT analysis were finalized.

## Results

### Strengths

**Innovation in Information Education and Communication (IEC):** Intensive and innovative IEC activities based on community inputs through various competitions was a highlight of the TSC in the district. Competitions in songs, essays, cartoons, scripts for street plays and posters were organized on the topic of open field defecation in which the community participated wholeheartedly. The contributions of the community formed the main content during the IEC campaign. The songs that won prizes were recorded into an audio cassette that was played in the villages during the IEC campaign. The best posters and cartoons were used for preparing display boards and hoardings that were displayed at the various block level offices. The best poems, songs, and essays were compiled into a book. The best slogans were used for preparing wall paintings. Thus, content of IEC activities was based on the original ideas and inputs of the local community.

**Motivation through incentives:** The awards under the campaign were important to motivate and mobilize the community. People were motivated not only by the cash award but also considered it a matter of pride and prestige. Apart from the awards, many other incentives were included in the campaign. After a village achieved Open defaecation free (ODF) status, the stakeholders were felicitated in the General Body meeting of Zilla Parishad (ZP). The names of all ODF villages were displayed on huge hoardings in the ZP compound. Women Self Help Groups (SHG) in which all members constructed a sanitary latrine were adjudged as a *Nirmal Bachat Gat*. Such SHGs were given priority for sanction of funds, training, etc.

**Competitive spirit:** The awards schemes inculcated a competitive spirit among the villagers. In some villages, this helped them overcome internal differences and work in unison to achieve the goal.

**Active participation and partnerships:** During the campaign, the community and the governmental agencies worked as partners. The government staff and officers took the initiative, attended the gramsabhas, and participated in IEC activities. The villagers dug pits and were willing to spend money for the construction of latrines. TSC proved that Panchayati Raj Institutions (PRI) can provide effective leadership and bring about social mobilization in villages.

**Women's role:** Women played important roles as beneficiaries, targets, and resources during the campaign. Women performed various roles as fund raisers, motivators, initiators, surveillance workers, and implementers. Women participation and leadership through SHGs, Grampanchayat membership, as well as teachers played an important role in TSC.

**Universal coverage:** Out of 416 households visited during the survey, 407 (97.84%) had individual household latrines, while members of 9 (2.16%) households were using community latrines. All 4 villages were free of open-field defecation. In the control villages, only 96 (17.94%) out of 535 households had individual household latrines while members of 439 (82.06%) households were practicing open-field defecation.

The campaign required that every household in the community stop open-field defecation and use a sanitary toilet. Therefore, this approach involved even the poorest households in the community and ensured that the community and local government focused on assisting these households in gaining access to adequate sanitation facilities.

**Sanitation ladder:** In one of the Nirmal Gram villages, the financial incentive given after achieving Nirmal Gram status with single pit latrines was utilized for construction of improved double-pit latrines. Building latrines facilitates a change in behavior wherein people give up the habit of open-field defecation. Once people get used to latrines, they improve on the type of latrines.

### Weaknesses

**No rural sanitary marts/production centers:** Currently there are no Rural Sanitary Marts (RSM)/ Production Centers (PC) in the district. Those which had been opened in the past had to be closed down. Neglecting this aspect can create a big gap between the requirement and supply of sanitation items.

**Women sanitary complexes:** A total of 53 Women Sanitary Complexes were constructed in the district. The poor maintenance and cleanliness of community latrines was also a weakness of the campaign.

**Targets affecting quality:** As the villages aimed to achieve the goal in a time-bound manner, in some cases, the latrines were built hurriedly to meet the target. A variety of superstructures were noticed. Some were made of plastic sheets, jute bags and tin sheets while some were made of cement and bricks.

**Monitoring and follow-up:** No agency was entrusted with the responsibility of follow-up and ensuring sustainability.

**Less focus on other aspects of sanitation:** The campaign mainly focuses on achieving ODF (Open Defecation Free) status wherein every individual in the village uses sanitary latrines leading to abolition of open field defaecation. During the field survey, it was observed that there was scope for improvement in disposal of sullage, solid waste, and animal waste [Tables 1 and 2].

**Table 1:** Sullage and solid waste disposal

Household waste	Methods	Nirmal Gram villages n = 416 (%)	Control villages n = 535 (%)
Sullage disposal	Soakage pit	226 (54.20)	48 (8.97)
	Open earthen unlined drain	0(0)	303 (56.64)
	Community garden	31(7.45)	9(1.68)
	Open concrete lined drain	159(38.35)	175(32.71)
Solid waste disposal	Composting	72(17.31)	57 (14)
	Burning	10 (2.4)	51 (12.53)
	Dustbin	215 (51.68)	120 (22.43)
	Open dumping	119 (28.61)	307 (57.38)

**Table 2:** Findings during the transect walks

Transect points	Nirmal Gram village	Control village
Solid waste disposal	Collected in public dust bins and burnt.	Open dumping.
	Vermi-composting units in some villages run by women self-help groups.	
Excreta disposal	Individual household latrines were in use and were clean. A variety of superstructures and latrines were cleaned by women in households.	Open field defecation. Separate areas allotted for men and women. Children sat in the courtyard and feces.
	Community latrines had poor maintenance and poor cleanliness.	disposed by open dumping.
	School and anganwadi latrines were in use and were clean.	School children use the space behind the school building.
Sullage disposal	Concrete lined open drains, some of which were blocked at places leading to water collection. Soakage pits, some of which were saturated.	Waste water flowing in earthen unlined open drains or along the slope leading to water collections near houses
	Household or community kitchen gardens.	
Animal waste disposal sites	Heaps of cow dung near houses collected for composting.	Cow dung lying along the roads.
	Compost pits in the fields.	

**Limitation of campaign approach as an entry point to overall rural development:** In the campaign approach, once the community decided to achieve the goal of Nirmal Gram, various internal and external forces conglomerated in time and place to achieve the goal expeditiously. The external factors like government support, guidance, IEC, training, monitoring, supervision and internal factors like spending money, labor and motivating others gave momentum to the program. The goal of total sanitation was usually achieved in a span of a few weeks for a village. But with this the focus of the external forces shifted to another village. Only a few activities related to sanitation e.g. vermicomposting or community gardens were taken up in two villages in the post campaign phase.. The opportunity for carrying forward the momentum to achieve overall village development was lost.

### Opportunities

**Tap additional resources:** There was scope for involving the health department in the campaign. The Male Multipurpose workers and Accredited Social Health Activist (ASHA) can play an important role in IEC and motivation. All the work related to village sanitation (i.e., selling hardware, masonry work, maintenance and repair, selling sanitary items like brooms, disinfectants, brushes, etc. and the responsibility of the collection of cattle dung, composting and vermiculture, cleaning drains, maintenance of community gardens and tree plantations) can be combined together and handed over to a SHG through a contract at the Grampanchayat level.

**Learn from experiences:** There is an opportunity to strengthen the campaign by incorporating the experiences from other projects e.g., the role of women SHGs, use of PRA (Participatory Rapid appraisal) techniques, etc.

**Establish a back-up agency at the district level:** Through TSC, the community proves its capacities and willingness to participate in development programs. The district administration can appoint a back-up agency that will continue to guide and support the villagers to achieve overall development.

**Overcome resistance:** During the field survey, the common reasons for not constructing latrines were a lack of space and money. Such resistance can be overcome by quoting and demonstrating examples from ODF villages where villagers had constructed latrines within their houses and purchased material in wholesale for the entire village or manufactured it at the village level.

### Threats

**Sustainability of interest and priority:** There is scope for expansion and increasing the coverage of the campaign. Sustaining the interest and priority accorded to the program at all levels of administration and in the community will be a challenge in the future.

**Changes in administration and leadership:** It is important to sustain the momentum and continue with the campaign irrespective of routine transfers of officers and staff and change in local leadership.

**Internal differences:** Achieving Nirmal Gram status is considered to be an important milestone by the village. It motivated groups in some villages to overcome internal differences while in other villages social and political differences came to the forefront during the campaign. There is a need to foresee and carefully handle such situations. The findings of the study are summarized in Table 3.

**Table 3:** SWOT analysis of Total Sanitation Campaign (TSC) in the district of Yavatmal

Strengths	Weaknesses	Opportunities	Threats
Innovations in IEC	No rural sanitary marts or production centers.	Tap additional resources.	Change in administration and local leadership.
Motivation through incentives	Poor maintenance of women sanitary complexes.	Learn from the experiences of other projects.	Sustaining interest and priority.
Competitive spirit	Targets affecting quality.	Develop back-up agency at the district level.	Internal differences.
Active participation and partnerships	Monitoring and follow-up.		
Women's role	Limitation of campaign approach as an entry point for overall rural development.	Overcoming resistance by quoting examples.	
Universal coverage	Less focus on other aspects of sanitation.		
Sanitation ladde	-		

IEC = Information Education and Communication; SWOT = Strengths weaknesses opportunities and threats.

## Discussion

The TSC is being implemented on specific principles with variations in approaches with respect to providing micro credit support, technical specifications, motivating the community, and ensuring sustainability.(4)

The campaign in Yavatmal was implemented along the principles and guidelines of TSC(5) with additional incentives and innovations. The interventions were successful in achieving ODF status for the entire village in a short span of time and can be replicated in other villages. Overall, the campaign aims at mainly achieving ODF status. The other components of sanitation need to be strengthened.

Innovation in IEC activities in the district was highly effective and can be replicated in other projects. The awards and competitive spirit have played a catalytic role in mobilizing the stakeholders and community. Once the goal of Nirmal Gram status is achieved, it is challenging to sustain the interest, collaboration, and cooperation in the campaign mode. Hence, as an entry point to overall rural development, this approach has some limitations. There is a need to identify back-up agencies and review, learn, and incorporate effective principles from other projects. The community-led TSC in Bangladesh based on PRA techniques has been found to be effective in this regard.(6)

The active role of PRI members and women has been reported in other districts.(7–9) Experiences in some states have revealed that SHGs can be a powerful local institution to manage sanitation and hygiene delivery.(10,11) Under National Rural Health Mission, the sanitation campaign has become an integral part of the district health plan and the institutional arrangements of TSC have been merged with other programs related to health.(12)

One of the major weaknesses in the campaign was the absence of RSMs and PCs. The main aim of having RSMs and PCs is to provide cost effective materials and guidance needed for the construction of different types of sanitary facilities that are suitable to rural areas. In an evaluation report, many RSMs were found to be lacking in financial sustainability and promotional activities.(13) It was noted that radical changes were necessary to make them viable and financially sustainable. The models of RSMs and PCs in some districts of West Bengal have been reported to be successful in this regard.(7,14) There is a need to plan the RSMs and PCs carefully considering the local situations and demands.

The concept of a sanitation ladder is important in the campaign.(15) The significance of the first cheap toilet is enormous in terms of breaking the habit of open defecation and getting people into the habit of using a latrine. The campaign was successful in overcoming internal differences in some villages while in other villages such differences proved to be a big hurdle. There is a need to foresee and handle the situation carefully to ensure success in the program. The responsibility of maintenance of community latrines needs to be clearly identified at the village level.

Improved sanitation contributes in the form of health, social, and economic benefits and will be an important step in ensuring the development and welfare of rural communities. A regular review of the program will help in identifying important factors that need to be strengthened, modified or rectified to ensure its success.
